# The Effect of Personality on Daily Life Emotional Processes

**DOI:** 10.1371/journal.pone.0110907

**Published:** 2014-10-24

**Authors:** Emma Komulainen, Katarina Meskanen, Jari Lipsanen, Jari Marko Lahti, Pekka Jylhä, Tarja Melartin, Marieke Wichers, Erkki Isometsä, Jesper Ekelund

**Affiliations:** 1 University of Helsinki, Department of Psychiatry, Helsinki, Finland; 2 University of Helsinki, Institute of Behavioural Sciences, Helsinki, Finland; 3 Hospital District of Helsinki and Uusimaa, Helsinki University Central Hospital, Department of Psychiatry, Helsinki, Finland; 4 Hospital District of Helsinki and Uusimaa, Helsinki University Central Hospital, Jorvi Hospital, Department of Psychiatry, Espoo, Finland; 5 Folkhälsan Research Center, Helsinki, Finland; 6 Vaasa Hospital District, Department of Psychiatry, Vaasa, Finland; 7 National Institute of Health and Welfare, Department of Mental Health and Substance Abuse Services, Helsinki, Finland; 8 Maastricht University, Department of Psychiatry and Psychology, School for Mental Health and Neuroscience, Maastricht, Netherlands; Southwest University, China

## Abstract

Personality features are associated with individual differences in daily emotional life, such as negative and positive affectivity, affect variability and affect reactivity. The existing literature is somewhat mixed and inconclusive about the nature of these associations. The aim of this study was to shed light on what personality features represent in daily life by investigating the effect of the Five Factor traits on different daily emotional processes using an ecologically valid method. The Experience Sampling Method was used to collect repeated reports of daily affect and experiences from 104 healthy university students during one week of their normal lives. Personality traits of the Five Factor model were assessed using NEO Five Factor Inventory. Hierarchical linear modeling was used to analyze the effect of the personality traits on daily emotional processes. Neuroticism predicted higher negative and lower positive affect, higher affect variability, more negative subjective evaluations of daily incidents, and higher reactivity to stressors. Conscientiousness, by contrast, predicted lower average level, variability, and reactivity of negative affect. Agreeableness was associated with higher positive and lower negative affect, lower variability of sadness, and more positive subjective evaluations of daily incidents. Extraversion predicted higher positive affect and more positive subjective evaluations of daily activities. Openness had no effect on average level of affect, but predicted higher reactivity to daily stressors. The results show that the personality features independently predict different aspects of daily emotional processes. Neuroticism was associated with all of the processes. Identifying these processes can help us to better understand individual differences in daily emotional life.

## Introduction

The relationship between personality and psychopathology is complex [1]–[4]. Neuroticism has been linked to a wide range of psychopathology [2], [4]. The association of extraversion is notably weaker and more equivocal [2], [3], [5]. In a recent meta-analysis, conscientiousness was strongly and negatively linked to psychopathology [2]. There are several alternative explanations for how personality is linked to psychopathology. Features of personality can predispose an individual to illness [6] and may be influenced by current [6] or previous psychopathology [7], [8].

Abundant evidence exists for two latent dimensions, internalizing and externalizing, underlying the structure and comorbidity of common mental disorders [9]–[11]. A recent study including epidemiological surveys from 14 countries found that the associations between a temporally primary mental disorder and another subsequent disorder were significantly stronger within than between internalizing and externalizing domains, and provided support for the existence of mediating latent internalizing and externalizing variables [11]. Neuroticism has been linked especially to internalizing, i.e. depressive and anxiety disorders, and their comorbidity in several studies [3], [7], [10], [12]–[14], and neuroticism shares genetic risk factors with internalizing disorders [5], [8], [12], [15]. Neuroticism has therefore been suggested to be one of the factors mediating risk for a spectrum of internalizing disorders [10], [16].

It has recently been argued that neuroticism is a non-specific and overly general trait [1], [4]. To better understand what neuroticism actually represents, Ormel et al. [17] have suggested “deconstructing” neuroticism by investigating its association with average level of affect separately from its association with reactivity of affect. Alterations in emotional life, especially experiencing strong and persistent negative affect, are closely related to internalizing disorders. Personality features are known to be associated in many ways with individual differences in emotional life, such as negative affect and affect reactivity. It is possible that this could be one of the factors mediating the link between personality features and psychopathology. For example experiencing higher negative affect and being more reactive to stressors in daily life could indicate vulnerability to internalizing disorders. Understanding what personality features indicate with respect to daily emotional processes could therefore help in future research to elucidate the poorly understood link between personality and mood and anxiety disorders.

Much literature exists on neuroticism’s association with negative affect and extraversion’s association with positive affect [18]–[22]. Negative affectivity is a basic and defining component of neuroticism [23]; however, in a recent study using multiple assessments per day, neuroticism was associated only with lower daily positive affect, not with higher daily negative affect [24]. Higher positive affect and lower negative affect have been associated with agreeableness [22], [25], [26] and conscientiousness [27], [28]. Moreover, while several studies have found no significant association with openness to experiences and positive and negative affect [22], [26], [28], a recent meta-analysis has linked this trait to higher positive affect [27].

Higher affect variability has been linked to neuroticism in several [24], [29], [30] but not all studies [26]. Agreeableness has been related to lower variability of affect [22], [26], whereas results for extraversion are ambiguous, with some studies linking it to lower variability [22] and others reporting no association [26], [30].

Personality features may also be associated with stress exposure. Individuals with high neuroticism tend to report more negative events in their daily lives [18], [31]–[33]. On the other hand, extraversion might protect from stressors and predict more positive daily life events [18].

A fourth aspect of daily emotional processes is affect reactivity. Neuroticism has been demonstrated to be associated with greater reactivity of negative affect to daily stressors in several studies using once-a-day diary reports [18], [31], [32], [34], [35] or momentary assessments conducted several times a day [36]. However, some studies, including a recent report applying the Experience Sampling Method [24], have not shown this association [37]. Extraversion has been observed to indicate higher reactivity to positive mood induction in some studies, but a notable number of contrary results also exist [38]–[40]. Furthermore, extraversion has been associated with increased amygdala responses to happy facial expressions and increased electrophysiological brain responses to positive stimuli, suggesting that extraversion could indicate enhanced sensitivity to reward [41], [42]. The question whether extraverts are less reactive to negative events has gained far less attention in personality research. A recent study however found increased brain responses in extravert individuals for highly unpleasant stimuli compared to neutral, but similar responses for moderately unpleasant stimuli and neutral ones [41]. Less extravert individuals on the contrary had increased brain responses also for moderately unpleasant stimuli, indicating extraversion to associate with lower sensitivity to negative stimuli, This could contribute to higher well-being of extraverts [21], [41]. In one study, agreeableness was associated with higher reactivity to interpersonal conflicts only [35].

As described above, the results from previous studies are somewhat mixed and partly contradictory, leaving still largely open the role played by personality features in daily emotional processes. The goal of this study was to explore the daily emotional processes in more detail and with high ecological validity. The study particularly aimed to dissect the daily emotional processes in four different categories; level of affect, affect variability, daily stressors, and affect reactivity, and to investigate how they are associated with different personality features. To our knowledge, this is the first study to investigate the association of the Five Factor personality features with different daily emotional processes separately in the same study. Previous studies, using multiple assessments per day [22], [24], [26], [28], [36] or a daily diary method [18], [31], [35], [43], have instead focused on just one or two personality features or emotional processes.

Our primary hypothesis was that neuroticism would predict higher negative and lower positive affect, higher affect variability, more reported negative daily incidents, and higher reactivity to these incidents. Also, by definition, neuroticism is related to negative affect, being vulnerable, unstable and reactive and having poor coping skills [44], [45]. Based on consistent existing research, we expected extraversion to be associated with a higher level of positive affect [18]–[21]. Although less consistent, previous research also supported an additional hypothesis that agreeableness and conscientiousness would predict higher positive and lower negative affect [25]–[28].

## Materials and Methods

### Subjects

Participants comprised 106 university students aged 19–35 years. The subjects were screened with the Structured Clinical Interview for DSM-IV Axis I Disorders [46] to rule out any current psychiatric disorders. One subject was excluded because of a current major depressive episode and one because of an insufficient amount of data (see below), thus resulting in a final sample of 104 subjects (18 males, 86 females). The mean age of subjects was 23 years (range 19–35, SD 3.69). They had been high achievers in comprehensive school (grade point average in the highest decile, 9.1 (SD 0.56) on a 4–10 scale, population average 7.5 (SD 0.94) (population average from personal communication with Dr. Juhani Rautopuro, Finnish National Board of Education)).

### Ethics Statement

The study was approved by the local ethics committee (Ethics Committee of Helsinki and Uusimaa Hospital District). Oral consent was obtained after careful oral and written information about the study protocol to the participants by the first author and overseen by the principal investigator. Since this study entailed healthy, adult individuals and the study procedures did not infringe on the integrity of the subjects, did not include any sample taking or administration of any substances but rather was observation of the subjects in their normal daily routines, written consent was not required by the ethics committee.

### Study Design

Momentary experiences of the subjects were collected for one week using the Experience Sampling Method (ESM). ESM is a momentary assessment method where subjects’ reports are collected randomly in response to a signal of an electronic device [47], [48]. ESM has been shown to be a valid and reliable method [47], [49]. Its advantages include the collection of a large number of reports for each subject, the lack of recall bias since it uses real-time assessments, enabling investigation of variation over time, and better ecological validity since it occurs in a natural environment [48], [50]. For the first half of the sample (51 subjects), the small portable PsyMate device (PsyMate B.V., Maastricht, Netherlands) was used to collect data. For the second half of the sample (53 subjects), an Android smart phone application (OLO ©), developed in-house for momentary assessment purposes, was used. The application worked analogically with the PsyMate. Both were programmed to beep 10 times per day at semi-random intervals (maximum time between beeps 4 h, minimum 15 min) between 7∶30 AM and 10∶00 PM. The subjects were instructed to use the device for 7+/−1 day. At each beep, the device presented a series of questions in a multiple-choice format about current emotions, activities, social context, and events since the last beep. The questions had to be answered within 15 minutes of the beep to ensure real-time assessment. The subjects answered the questions using the touch screen of the device. They were asked to continue their normal lives without changing their daily routines and to keep the assessment device with them at all times. Consistent with previous ESM studies, to include the participant in the data set we required a response rate of at least one in three beeps since this has been shown to be a valid cut-off for reliability [24].

### Measures

#### Daily life affect

We assessed momentary affective states using words adapted from Russell’s circumplex model of affect [51]. The words selected formed a circumplex with two dimensions, valence and arousal [52]. Negative affect words were the Finnish translations of sad [surullinen] (neutral arousal), nervous [hermostunut] (high arousal), and tired [väsynyt] (low arousal). Positive affect words were cheerful [iloinen] (neutral arousal), excited [innostunut] (high arousal), and content [tyytyväinen] (low arousal). We used the word active [aktiivinen] to measure a high arousal affective state with neutral valence and tranquil [rauhallinen] to measure a low arousal affective state with neutral valence. The subjects reported their current emotions by answering a question, e.g. “Right now I feel sad”, using a 7-point Likert scale (0 = not at all, 6 = extremely).

#### Affect variability

In line with previous studies, we used the standard deviation of each affect as a measure of affect variability [24], [30].

#### Context and affect reactivity

The subjects reported their current activities (“What were you doing just before the beep?”), events since the last beep (“What was the most important event since the last beep?”), social context (“Who are you with right now?”), and subjective evaluations of these activities.

The sum of the subjective evaluation items “I enjoy this activity” and “I can do this well” (0 = not at all true, 6 = completely true) formed the “quality of activity” variable.

The question referring to the most important event since the last beep “How pleasant was the event” (0 = very unpleasant, 6 = very pleasant) was used as the “quality of event” variable.

Social context was first evaluated with a question about whether the subject was alone or not. If a subject responded not being alone she was next asked if she would prefer being alone (“I would prefer being alone”, 0 = not at all true, 6 = completely true). This was used to assess the subjective “quality of social interaction” from highly negative (6) to highly positive (0) (i.e. if a subject was in company but preferred being alone at the moment, this was interpreted as low quality of social interaction). If the subject answered that she was alone, the next question was whether being alone was by choice (0 = not at all true, 6 = completely true). This response was used in our analyses to assess “quality of solitude” from highly negative (0) to highly positive (6).

As in previous studies [18], [24], [53], [54], we assessed affect reactivity by the negative affect response to daily life contexts.

#### Personality traits

The Five Factor personality traits were measured using a Finnish version of NEO Five Factor Inventory (NEO-FFI) [55], [56], which the participants completed before being given the assessment device. The questionnaire is answered with 5-point likert scale (e.g. “I am not a worrier”, “I often feel tense and jittery”, “I work hard to accomplish my goal”, 1 = strongly disagree, 5 = strongly agree).

### Statistical Analysis

Hierarchical linear modeling (HLM), more specifically linear growth curve models, was used (e.g. [57], [58]). HLM takes into account the hierarchical structure of the data, i.e. dependencies of the data on a within-subject level and on repeated measurements. It also allows an unequal number of repetitions. The data were analyzed with MIXED procedure in the IBM SPSS Statistics software, version 21 (IBM Corporation, Armonk, New York, USA). Time of response was used as a level 1 predictor and personality traits or daily contexts as a level 2 predictor. Both level 1 and 2 predictors were fixed, but the parameters (intercept and slope) of the time variable (level 1 predictor) were allowed to vary randomly between subjects. All variables were standardized to allow comparison of the regression coefficients with each other. The outcome, B, is the standardized regression coefficient of HLM and can be interpreted analogously to standardized coefficients in standard regression analyses. All models were adjusted for gender, age, and assessment device used (PsyMate or OLO).

In Model 1, the effect of personality traits on level of affect was analyzed using each emotion as a dependent variable and traits as predictors. In Model 2, the effect of personality on daily incidents was analyzed using subjective evaluations of events, activities, social interaction, or solitude as dependent variables. In Model 3, affect reactivity was assessed. Sadness was selected as a dependent variable to reflect reactivity of negative affect because in Model 1 the personality traits had the greatest effect on sadness of all emotions (see results). In the reactivity model, both traits and context evaluations were used as predictors, and the statistical interaction of trait and context evaluation was used to assess reactivity.

The effect of personality traits on affect variability was evaluated using bivariate correlation analysis. The standard deviation was first calculated for each subject and each emotion (across all responses of each subject). The correlations of personality traits with standard deviations were then calculated.

## Results

The participants responded to 54 beeps on average (SD 9.87, range 28–94). The total number of beeps responded to across all subjects included in the final data was 5599. The descriptive statistics of the ESM measurements and the personality features measured by the Five Factor Model are shown in Tables 1 and 2.

**Table 1:** Descriptive statistics for ESM measurements and Five Factor personality features.

	Mean	SD	Min	Max
Cheerful	3.81	0.59	1.82	5.59
Content	3.82	0.57	2.56	5.37
Excited	3.06	0.64	1.67	4.68
Sad	0.77	0.67	0.00	2.79
Tired	2.54	0.83	0.74	4.72
Nervous	1.23	0.75	0.05	3.54
Calm	3.82	0.65	2.07	5.21
Active	3.05	0.65	1.24	4.66
Beeps alone	18.80	9.33	3.00	45.00
Quality of event	4.40	0.48	3.39	5.70
Quality of activity (I enjoy this)	4.11	0.55	3.12	5.68
Quality of activity (I can do this well)	4.68	0.64	3.32	5.96
Quality if social interaction	4.97	0.70	2.96	6.00
Quality of solitude	4.20	1.61	0.00	6.00
Neuroticism	1.34	0.59	0.17	3.25
Extraversion	2.46	0.42	1.16	3.58
Openness	2.66	0.55	1.17	3.75
Agreeableness	2.89	0.46	1.33	3.75
Conscientiousness	2.65	0.61	1.25	3.92

*Note*: The means are aggregated over the means of the participants. “Beeps alone” is the number of responses while being alone. Quality of social interaction has been re-coded from the original item for the sake of clarity of the table, with higher rate referring to more positively evaluated social interactions.

**Table 2:** Proportion of each activity or event of all beeps across all subjects.

	Most important event since last beep (%)	Current activity (%)
Work/studying	11.9	17.7
Physical activity	7.7	6.6
Media/reading	8.2	17.3
Passive/doing nothing	5.4	8.9
Chores	10.2	14.8
Social interaction	34.9	21.6
Eating	11.8	5.0
Something else	9.3	7.8

### Effect of Personality on Daily Life Level of Affect

The results of Model 1 are presented in Table 3. Neuroticism was significantly associated with higher average daily sadness and nervousness and lower positive affect as well as lower activity and calmness. Agreeableness was significantly associated with higher cheerfulness, contentment, and calmness and lower negative affect. Conscientiousness predicted lower sadness and nervousness as well as higher activeness. Extraversion predicted higher cheerfulness and excitement, but showed no significant associations with negative affect. Openness did not have any significant associations with momentary affect.

**Table 3:** Standardized regression coefficients from Model 1.

	Cheerful	Tired	Content	Nervous	Calm	Sad	Excited	Active
Neuroticism	−0.15***	0.08	−0.18***	0.16***	−0.15**	0.24***	−0.14***	−0.13**
Extraversion	0.15***	−0.01	0.11**	−0.04	0.08	−0.05	0.11*	0.07
Openness	0.02	−0.05	0.01	0.05	−0.07	0.06	0.03	−0.01
Agreeableness	0.12**	−0.10*	0.16***	−0.12*	0.14**	−0.15**	0.07	−0.04
Conscientiousness	0.04	0.03	0.06	−0.10*	0.09	−0.15**	0.06	0.08*

Hierarchical linear modeling with each affect as a dependent variable and personality trait as a predictor.

*Note:* *p≤.05, **p≤.01, ***p≤.001. The model was adjusted with gender, age, and assessment device used and includes only main effects of the variables.

### Effect of Personality on Affect Variability

Correlation analysis between variability (standard deviation) of each affect and the personality traits (Table 4) revealed that neuroticism correlated with higher variability of contentment, nervousness, sadness, and excitement, agreeableness with lower variability of sadness, conscientiousness with lower variability of sadness and nervousness, and openness with lower variability of activeness.

**Table 4:** Correlations of the personality traits and standard deviations of the momentary affect across all assessments aggregated over each participant.

	Cheerful	Tired	Content	Nervous	Calm	Sad	Excited	Active
Neuroticism	.16	.08	.22*	.25*	.08	.30**	.20*	.16
Extraversion	−.07	.03	−.11	.01	.07	−.02	−.09	−.13
Openness	−.11	−.16	−.12	.02	−.13	.04	−.11	−.20*
Agreeableness	−.06	−.12	−.01	−.08	−.02	−.24*	−.07	.04
Conscientiousness	−.10	−.02	−.11	−.22*	−.04	−.21*	−.10	−.16

*Note:* *p≤.05, **p≤.01, ***p≤.001.

### Effect of Personality on Daily Life Contexts

The results of Model 2 are presented in Table 5. More positively evaluated daily events and activities were negatively associated with neuroticism and positively with agreeableness. Agreeableness was also associated with more positively evaluated social situations. Moreover, extraversion and conscientiousness were associated with more positively evaluated activities, and openness with more positively evaluated solitude.

**Table 5:** Standardized regression coefficients from Model 2.

	Quality of event	Quality of activity	Quality ofsocial interaction	Quality ofsolitude
Neuroticism	−0.08**	−0.18***	−0.003	0.01
Extraversion	0.05	0.11*	−0.02	−0.01
Openness	−0.04	−0.001	0.05	0.19**
Agreeableness	0.08**	0.13**	0.15***	0.03
Conscientiousness	0.03	0.09*	0.01	−0.06

Hierarchical linear modeling with each context evaluation as a dependent variable and personality trait as a predictor.

*Note:* *p≤.05, **p≤.01, ***p≤.001. The model was adjusted with gender, age, and assessment device used and includes only main effects of the variables.

### Effect of Personality on Affect Reactivity

Model 3, the reactivity model, was built to investigate whether the effect of daily stressors on the level of sadness depends on the personality traits, i.e. whether there is a statistical interaction between trait and stressor on the level of negative affect. In order to make the regression coefficients more intuitive, the results are reported as the interaction of personality traits and *lower* quality of daily incidents (i.e. daily stressors) on the level of sadness.

**Figure 1:**
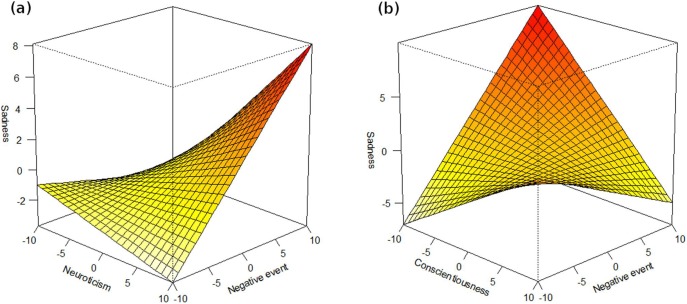
The association of (a) neuroticism and (b) conscientiousness with reactivity to daily negative events. The figure illustrates Hierarchical linear modeling with sadness as a dependent variable and (a) neuroticism or (b) conscientiousness and negatively evaluated daily event as well as their interaction as a predictor.

Neuroticism predicted higher reactivity of sadness to all of the assessed daily stressors (see results in Table 6, see Figure 1 for illustration of selected associations). We found conscientiousness to predict lower reactivity to all of the daily stressors except negative solitude. Agreeableness predicted lower reactivity only to negatively evaluated social interaction. Openness predicted higher reactivity to most of the daily stressors.

**Table 6:** Standardized regression coefficients from Model 3.

		Negative event	Negative activity	Negative social interaction	Negative solitude
Neuroticism	B_trait_	0.22***	0.20***	0.24***	0.23***
	B_stressor_	0.19***	0.18***	0.18***	0.15***
	B_interaction_	0.02*	0.04***	0.05***	0.08*
Extraversion	B_trait_	−0.04	−0.03	−0.06	−0.02
	B_stressor_	0.20***	0.19***	0.18***	0.16***
	B_interaction_	0.02	−0.01	−0.003	−0.04
Openness	B_trait_	0.05	0.06	0.09	0.02
	B_stressor_	0.20***	0.19***	0.20***	0.16***
	B_interaction_	0.01	0.03**	0.06***	0.08**
Agreeableness	B_trait_	−0.13*	−0.12*	−0.13*	−0.12
	B_stressor_	0.20***	0.19***	0.17***	0.16***
	B_interaction_	−0.01	−0.02	−0.04***	−0.01
Conscientiousness	B_trait_	−0.15**	−0.14**	−0.14**	−0.14*
	B_stressor_	0.20***	0.19***	0.18***	0.16***
	B_interaction_	−0.05***	−0.05***	−0.04**	−0.04

Hierarchical linear modeling with sadness as a dependent variable and each personality trait and stressor as well as their interaction as a predictor.

*Note:* B_trait_ and B_stressor_ are the main effects of personality traits and daily stressors on the level of sadness. B_interaction_ is the effect of reactivity, i.e. interaction of personality trait and stressor on the level of sadness. “Stressors” refer to negatively evaluated daily events/activities/social interactions/solitude. *p≤.05, **p≤.01, ***p≤.001. The model was adjusted for gender, age and assessment device used.

The effect of being alone per se, without taking into account the subjective evaluation of the situation, was also tested, but no significant associations with personality features emerged.

## Discussion

Our findings provide new information about the associations of the Five Factor personality traits with affective reactivity in daily life. Moreover, our findings contribute to the current knowledge of the associations between personality traits and average level of affect, affect variability and daily stressors.

The results for neuroticism were consistent with our primary hypothesis. They indicate that neuroticism not only is associated with the average level of affect, but has an impact on several processes of daily emotions. Most importantly, our findings support the somewhat debated view that neuroticism indicates higher affect reactivity in daily life [36]. The association of neuroticism with emotional reactivity has an intriguing link to depression. Previous studies have shown that neuroticism amplifies the depressogenic effect of stressful life events, i.e. the interaction of stressful life events and neuroticism predicts onset of depression [59], [60]. A potential mediator for this could be the effect that neuroticism has on emotional reactivity to stressors, as observed here. Neuroticism not only exposes to stressors but indicates vulnerability to them in daily life. Based on our findings, we cannot discern whether neuroticism actually influences the amount of stressors or just the subjective evaluation of them, but we can state that more neurotic persons report more negative daily events and activities. Recurrent negative experiences can reinforce negative attributions and appraisals and vice versa; negative attributions and appraisals, as well as higher average level and reactivity of negative affect, can make a person more prone to stress exposure. Negative bias in information processing [61] as well as negative appraisal styles [28] have previously been linked to neuroticism. This combination of negative affective processes could predispose persons with high neuroticism to internalizing disorders.

Conscientiousness, as opposed to neuroticism, was the only trait that predicted lower reactivity to most of the assessed daily stressors. Expectedly, it also predicted a lower average level of negative affect. A recent meta-analysis showed conscientiousness to be the second most powerful trait correlating with internalizing disorders[2]. It has been argued that at least part of this correlation could be explained by the effect of psychopathology on personality since especially affective disorders can cause demoralization and negative self-efficacy, which can lead to lower conscientiousness scores [2]. In our study the subjects were healthy young students without psychopathology, which indicates that the association of conscientiousness with emotional processes exists in the absence of any psychiatric illness. We suggest the possibility that the emotional processes mediating the link between personality features and internalizing disorders could be a combination of high negative affect and high reactivity to daily stressors, which in our study was associated with neuroticism. The opposite combination, which in our study was associated with conscientiousness, could be protective. This proposition is speculative and investigating it empirically would require a different study design, preferably longitudinal and comparing healthy subjects to patients. However, our results do provide a new approach for investigating personality and etiology of psychiatric illnesses. One approach to “deconstruct” personality traits, as suggested by Ormel et al. [17], is to consider them as combinations of differentially biased emotional processes that can be more specific vulnerability markers than the Five Factor traits themselves. It would be interesting to specifically investigate if, and how these combinations of daily emotional processes are associated with psychopathology, and if they can explain the link between personality and psychopathology.

This could also help to clarify the association of extraversion with depression, which, based on previous studies, remains modest and somewhat equivocal [2]. It has been suggested that in fact the positive affect facet of extraversion, but not the other facets, is linked to depression [62]. A higher level of positive affect has also been shown to decrease the effect of genetic vulnerability to depression [63]. In this study, we observed, as expected, the association of extraversion with higher positive affect, but not with negative affect or reactivity.

Agreeableness appeared to be specifically protective against the effect of social stressors, but not against other kinds of stressors (i.e. events, activities, and solitude). Persons with high agreeableness tend to appraise social situations more positively, and the trait seems to protect against the effect of stressful social situations on affective state. This can be a highly important and adaptive characteristic in modern society, which requires handling various social situations and values good social skills. Agreeableness has indeed been linked to happiness [27] and positive affect [25] as strongly as extraversion. Interestingly, however, a meta-analysis [2] found no significant link between agreeableness and psychopathology.

The same meta-analysis [2] found no correlation between openness and psychopathology. In our study, we did, however, observe an association between openness and higher reactivity to daily stressors. Openness, by definition, is related to being imaginative, esthetically reactive, creative, and artistic [45], characteristics close to emotional sensitivity. In this sense, it is intuitively feasible that openness could also increase reactivity to daily stressors. Since openness does not have an impact on average level of negative affect, but only on reactivity, it could be speculated that reactivity as indicated by openness is adaptive, enhancing creativity and imaginativeness.

An important strength of our study is the ecological validity of the Experience Sampling Method, which allows real-time experiences in everyday life to be collected. The lack of recall bias is especially important because of the possible negative information bias related to neuroticism [61]. Using an electronic assessment device ensures real-time assessment and decreases the risk for recall bias relative to paper-and-pen ESM studies. The most important limitation of our study is that the study sample included only young, mostly female students who had been high achievers in school. This hinders generalization of the results to the general population. Evidence does exist about gender-specific genetic effects on neuroticism [10] and therefore our results cannot directly be generalized to male population. However, the study sample can also be seen as a strength since, as stated earlier, it enables evaluation of the emotional processes of healthy persons without the confounding effect of psychopathology. A uniform sample also minimizes other causes of variance, and therefore the observed effect is more likely due to personality differences. We assessed the effect of each personality dimension independently. Naturally, there can be some degree of correlation and/or interaction between the dimensions. Specifically, extraversion and neuroticism have been found to interact to predict positive and negative affect [64]. We observed no such interaction between extraversion and neuroticism, and this subject was not investigated further.

## Conclusion

Our results indicate that personality features can influence several different daily emotional processes, i.e. average level, variability, subjective evaluation of daily incidents, and reactivity. Identifying these processes can shed light on individual differences in daily emotional life. This could help in the efforts to elucidate the link between personality and psychopathology. Our study, utilizing an ecologically valid method, shows that neuroticism has the broadest associations of all five features with daily emotional processes, predicting not only higher average level but also higher reactivity of negative affect. Conscientiousness predicted mostly the opposite. We suggest that reactivity of negative affect could have specific importance in terms of the link between personality features and depressive and anxiety disorders.

## Supporting Information

Dataset S1 (SAV)
